# Global flood extent segmentation in optical satellite images

**DOI:** 10.1038/s41598-023-47595-7

**Published:** 2023-11-20

**Authors:** Enrique Portalés-Julià, Gonzalo Mateo-García, Cormac Purcell, Luis Gómez-Chova

**Affiliations:** 1 Image Processing Laboratory, University of Valencia, Valencia, Spain; 2 Trillium Technologies, 27-29 South Lambeth Rd, London, SW8 1SZ UK; 3 School of Computer Science and Engineering, University of New South Wales (UNSW), Sydney, Australia

**Keywords:** Natural hazards, Computational science, Scientific data, Imaging and sensing

## Abstract

Floods are among the most destructive extreme events that exist, being the main cause of people affected by natural disasters. In the near future, estimated flood intensity and frequency are projected to increase. In this context, automatic and accurate satellite-derived flood maps are key for fast emergency response and damage assessment. However, current approaches for operational flood mapping present limitations due to cloud coverage on acquired satellite images, the accuracy of flood detection, and the generalization of methods across different geographies. In this work, a machine learning framework for operational flood mapping from optical satellite images addressing these problems is presented. It is based on a clouds-aware segmentation model trained in an extended version of the WorldFloods dataset. The model produces accurate and fast water segmentation masks even in areas covered by semitransparent clouds, increasing the coverage for emergency response scenarios. The proposed approach can be applied to both Sentinel-2 and Landsat 8/9 data, which enables a much higher revisit of the damaged region, also key for operational purposes. Detection accuracy and generalization of proposed model is carefully evaluated in a novel global dataset composed of manually labeled flood maps. We provide evidence of better performance than current operational methods based on thresholding spectral indices. Moreover, we demonstrate the applicability of our pipeline to map recent large flood events that occurred in Pakistan, between June and September 2022, and in Australia, between February and April 2022. Finally, the high-resolution (10-30m) flood extent maps are intersected with other high-resolution layers of cropland, building delineations, and population density. Using this workflow, we estimated that approximately 10 million people were affected and 700k buildings and 25,000 km$^2$ of cropland were flooded in 2022 Pakistan floods.

## Introduction

Floods are one of the most destructive and frequent extreme events that exist: between 1995 and 2015, 2.6 billion people were affected by floods, accounting for 56% of people exposed to weather-related disasters^1^. A large portion of the exposed people (89%) live in low-to-middle income countries^2^ where the effects of the floods are sometimes followed by a food security crisis^3^. According to Tellman et al.^4^, the population affected by floods grew by 58-96 million from 2000 to 2015 and the frequency and magnitude of flood events have also increased. Climate change projections indicate that these trends will continue^4–7^. In 2022, two striking flooding events hit Pakistan and Australia, both of them cataloged as very high-return events and the worst in their countries in a century. In Australia, heavy rainfall episodes in February, March, and April broke all historical records in several main cities in the eastern part of the country. Thousands of houses and businesses were flooded with an estimated cost of claims of 1.83$ billion USD^8^. Persistent heavy rainfall throughout the remainder of 2022 led to further extreme flooding events in October, affecting Queensland, NSW and Tasmania. In Pakistan, two months of heavy monsoonal rains affected 33 million people and caused very serious food security issues due to cropland losses with an estimated flood impact cost of 14.9$ billion USD in damage and 15.2$ billion in economic losses^9^.

Flood extent maps derived from satellite images are key to understanding the magnitude of a flooding event, as well as quantifying and mitigating its damage. In the first stage, flood extent maps are used for planning and assisting the emergency response on the terrain. In the second stage, these maps are used for flood damage assessment by intersecting them with high resolution geographical layers. Nowadays, with the proliferation of free and global high resolution map products, flood damage assessment can be carried out automatically in many locations worldwide. However, current approaches for operational flood extent mapping and damage assessment at a global scale present several limitations. Firstly, in order to cover large areas, typically low to medium resolution products ($\ge 100$ m) are used to produce flood extent maps. For instance, Tellman et al.^4^ estimated population exposure to flooding using MODIS products (250 m spatial resolution), which in urban areas with high population density may lead to very imprecise estimates^10^. Another example is the flood extent map released by UNOSAT for the flood event in Pakistan, which was generated using 375 m VIIRS data^11^, also used to derive statistics of the affected population and post-flood damage.

**Figure 1:**
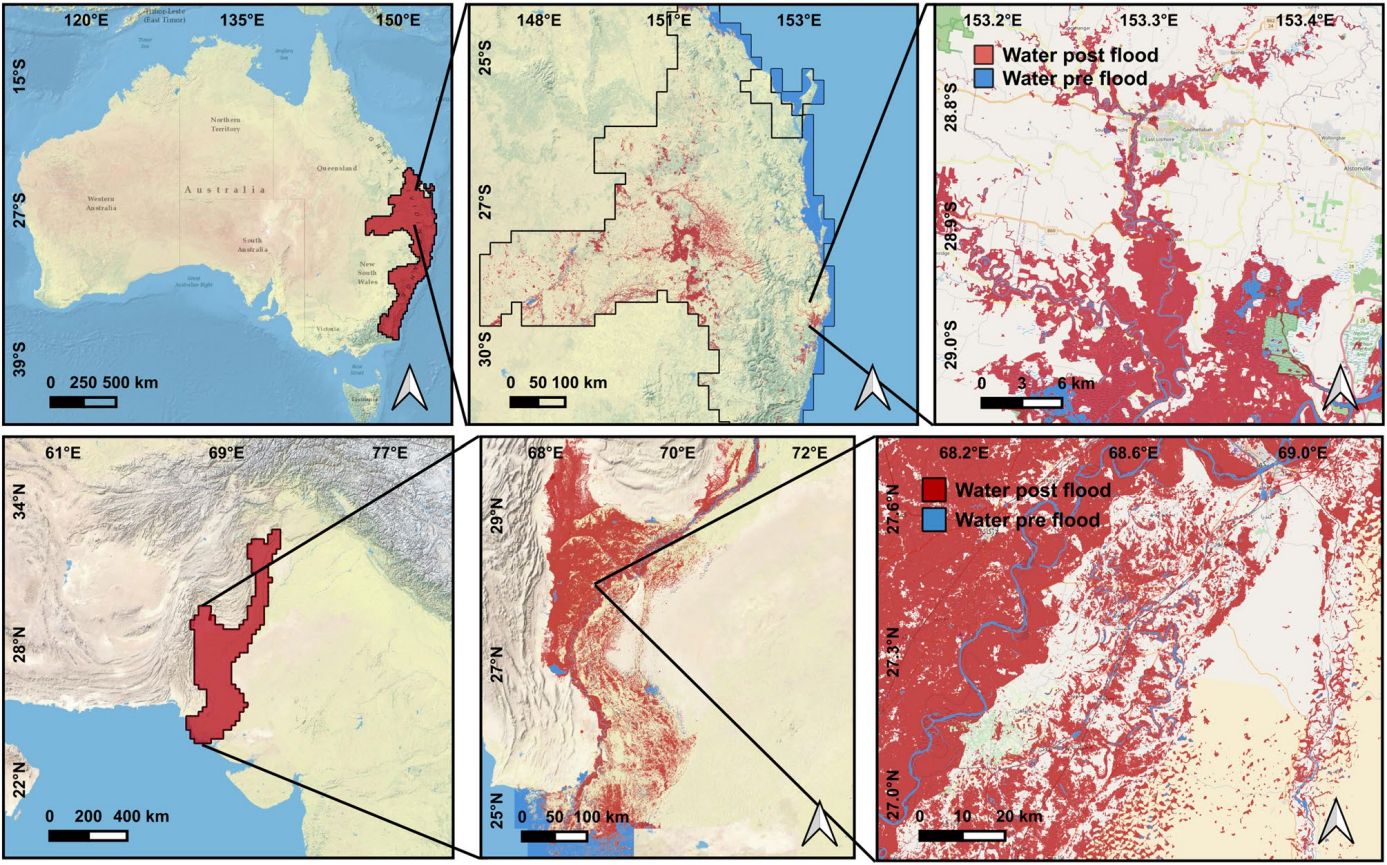
Vector-format flood extent maps generated through our pipeline for the Australia and Pakistan case studies. From left to right, the figures depict the entire Area of Interest under consideration, a section of the flood map, and a closer view of city-size region: the city of Lismore (Queensland) in Australia and Sukkur (Sindh province) in Pakistan, to showcase the precise resolution of the generated flood maps. These maps have been produced using Quantum GIS (QGIS)^12^.

Imagery from Sentinel and Landsat constellations can potentially be used for more accurate global flood extent mapping since they have a much higher spatial resolution (10 m and 30 m, respectively) and also a high revisit frequency (2–4 days depending on the geographic location)^13^. Additionally, flood extent maps based on cloud-free optical imagery, e.g. Sentinel-2, are more accurate than those based on Synthetic Aperture Radar (SAR) such as Sentinel-1^14^. This is because, inherently, SAR data for flood mapping faces challenges such as speckle noise interference, inconsistencies from wind-driven capillary waves, distortions from layover and shadow effects due to side-looking geometry, terrain-induced artifacts, and variations from polarization effects. In particular, accurately detecting floodwater becomes a greater challenge in urban environments or areas covered by dense forest canopies. In such scenarios, floodwater segmentation from optical imagery, while complex, tends to yield more successful outcomes^15^. However, there are several limitations that hamper the use of data from optical sensors in operational pipelines. First, clouds are very frequent in peak-flood and post-flooding imagery. In the WorldFloods dataset^16^, approximately 50% of the pixels are marked as cloudy in the first available Sentinel-2 revisit: on average 1.3 days after the date of the flood event^16^. Thus, in order to exploit partially cloudy revisits, flood detection algorithms should be able to operate in the presence of clouds and optimally distinguish between thin semitransparent clouds, where the surface can still be observed, and opaque thick ones^17,18^. Secondly, most methods applied automatically to large scale flood events are based on spectral indices that exploit the spectral properties of water bodies^4,19–22^. These methods have been widely studied and used in the remote sensing community, but they produce a significant amount of false positives in dark surfaces and situations, such as high sun zenith angles, dark soils, terrain shadows, and cloud shadows^23,24^. Additionally, these methods are reported to underperform when the water has suspended debris or pollutants, which are very frequent in flooding episodes^25^; and they do not properly capture flood traces, i.e. non-flooded areas in the acquired satellite image that were inundated in the peak of the event. Thirdly, potentially more accurate solutions, such as those based on machine learning, lack comprehensive datasets for training the detection models and are validated on a few flooding episodes over a limited number of different geographies. In addition, given the more complex nature of these models, most of them are not open-source, nor are the final trained models available, which operational application and intercomparison very difficult^26^.

In this work, we address these limitations by demonstrating an end-to-end flood mapping system to produce flood extent maps from Sentinel-2 and Landsat-8/9 optical imagery without human intervention. Our pipeline uses a new cloud-aware flood segmentation model that produces independent cloud and water masks, and it is able to detect flood water under semitransparent clouds. The model is carefully validated in a manually labeled dataset of 11 globally distributed flood events.

In order to demonstrate the capabilities of the system for large scale flood extent mapping, we run the proposed pipeline over the extreme flooding events in Australia and Pakistan in 2022 (Fig. 1) to produce vectorized flood extent maps covering a total area of 475,000 km$^2$ and 165,000 km$^2$, respectively. Afterward, we derive flood damage maps and metrics by intersecting the flood extent maps with global high-resolution products of population density^27^, buildings^28^, and crops^29^. We publish both the dataset and code, which are accessible at ml4floods (https://spaceml-org.github.io/ml4floods), a comprehensive Python package that covers data ingestion and processing, model training and evaluation, inference and all the necessary tools to post-process the results to derive vectorized flood products.

## Results

### Flood mapping and damage assessment in large flooding events

#### Pakistan floods

Pakistan monsoon season started in late June 2022 and lasted until late September 2022. During this period several episodes of prolonged torrential rains occurred, resulting in 375 mm of rainfall, almost three times higher than the national 30-year average of 130.8 mm^30^. This fact, preceded by a period of prolonged drought, resulted in one of the most catastrophic events that the country has ever experienced. For these reasons, there have been great efforts to map the extent of this event ^11,31^, and to develop new frameworks to quantify its consequences^32^.

We ran our flood segmentation method on imagery acquired by Sentinel-2 (S2) and Landsat 8 and 9 (L8/L9) to produce a vectorized flood extent map with pre-flood and post-flood water polygons. To create this map, we defined a region of interest covering the Indus river basin and we split this region into a grid of square tiles of 25$\times$25 km$^2$. For each of the tiles, we downloaded pre-event images from the 16th of May to the 6th of June, and post-event cloud-free images from the 25th of August to the 10th of September. In total, we processed 2491 Sentinel-2 images and 1691 Landsat images, corresponding to 265 GB of data acquired over an area of 165,000 km$^2$.

**Figure 2:**
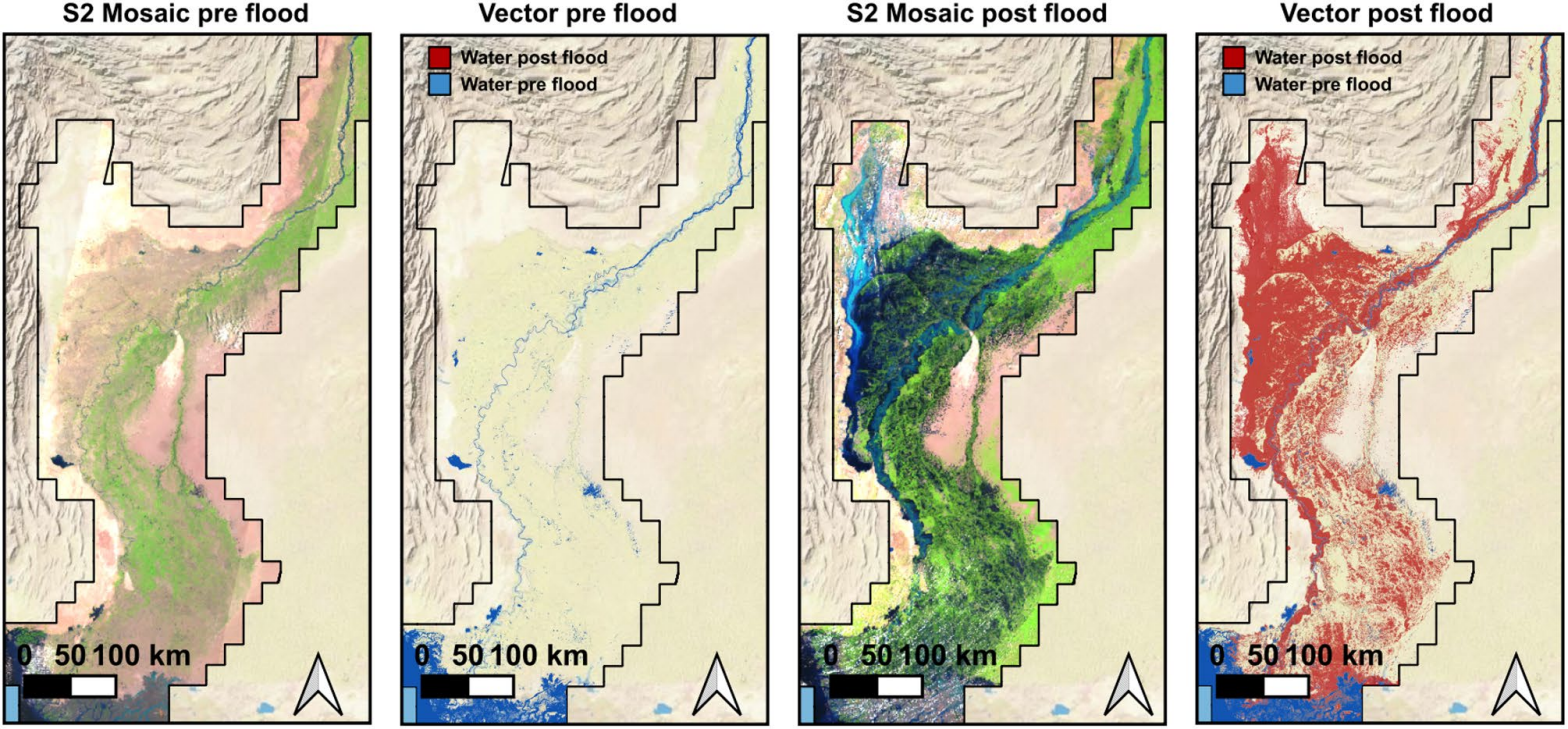
Vector flood product of Pakistan floods in 2022. False color composites of Sentinel-2 multispectral bands (B11, B8, B4) were produced with images from early June (pre-event composite) and early August (post-event composite). The considered area of interest covers an area of 165,000 km$^2$. In total, 265 GB of Sentinel-2 and Landsat imagery were used to produce these flood products. These maps have been produced using Quantum GIS (QGIS)^12^.

**Figure 3:**
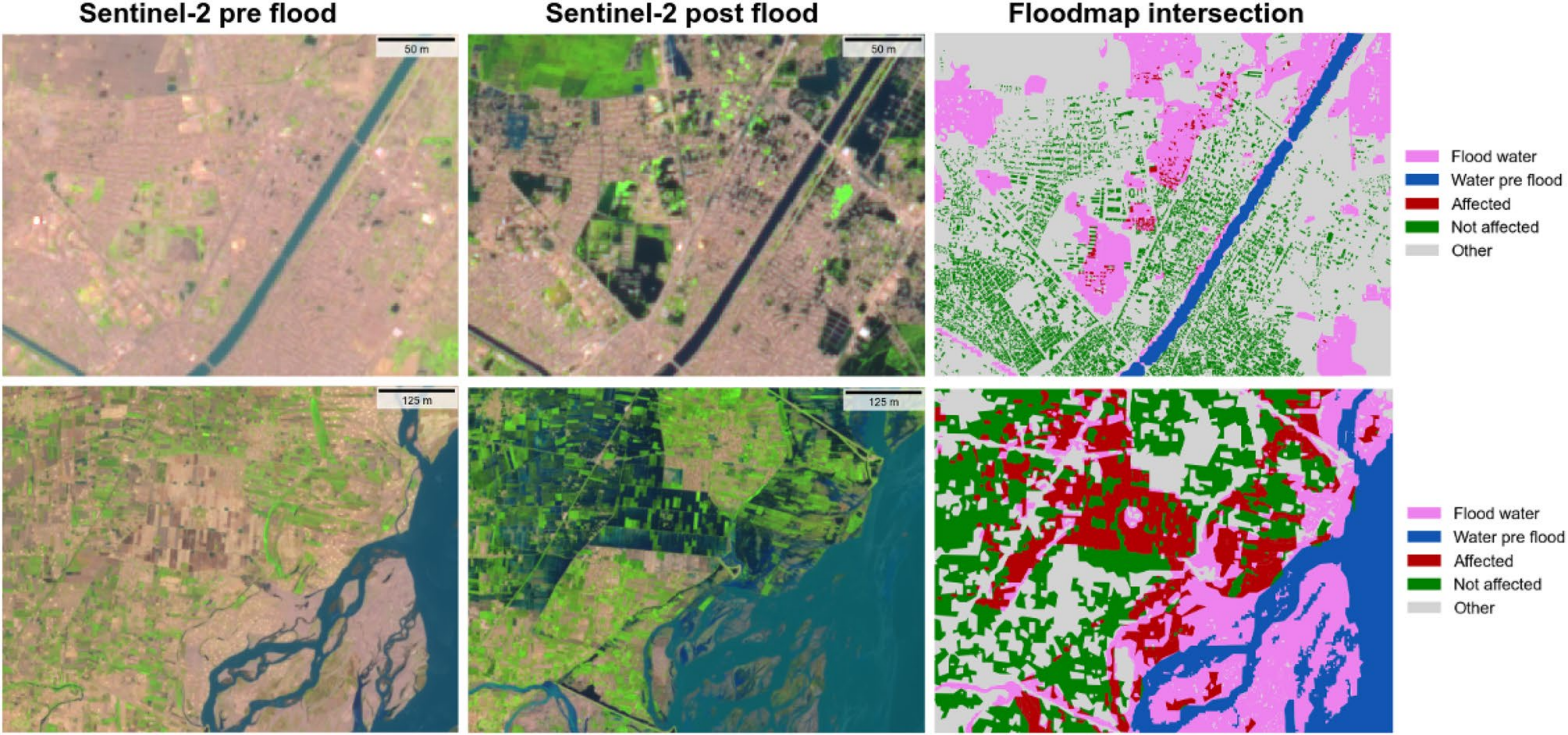
Example of the intersection of the flood product with selected land-cover and building delineation products. In the top row, we examine Larkana, a major city in Sindh province. Here, our estimation is that 36,000 buildings and approximately 240,000 people were potentially affected by the flood within this grid tile. In the bottom row, our focus shifts to an agricultural area near Layyah, located in the Punjab region. In this grid tile, the intersection indicates an estimated 107 km$^2$ of potentially damaged cropland.

We run our segmentation model in all the downloaded images producing per-pixel maps (raster masks) with three classes: water, cloud, and land. In this process, we vectorize the water and cloud masks, aggregate these vector flood maps in time, and mosaic them. For the temporal aggregation, we first obtain pre-event (permanent) water from the pre-event maps. Afterward, we derive the post-event product by merging the time series of post-event maps obtained for each grid tile, resulting in a maximal flood extent product. After obtaining the flood extent product for each individual grid tile, we perform a spatial merge over all the tiles to obtain the complete flood map. Finally, we subtract the pre-event product from the post-event flood map, resulting in the final flood inundation product that distinguishes between pre-flood and post-flood water. Figure 2 shows the pre- and post-event mosaics with the Sentinel-2 imagery and the final flood maps. Although extremely large in spatial extent, the final flood map has been produced with multispectral images of 10m and 30m resolution, allowing flood damage assessment at these fine spatial scales. For damage quantification, we compute the affected population, damaged infrastructure, and areas of cropland lost. For estimating the damaged infrastructure, we use the Microsoft Building Footprints^28^, which provides worldwide building delineations derived from very high-resolution Maxar images; for cropland impact assessment, we vectorize the ESA Land Cover Map from 2020^29^; and for the affected population, we use the High Resolution Settlement Layer (HRSL)^27^. Figure 3 shows an example of pre- and post-flood Sentinel-2 images and the intersection of the flood map with those layers. In the top row, we show a pre-flood (27th June 2022) and post-flood (31st August 2022) image covering the city of Larkana, one of the largest in Pakistan, and the intersection of the flood extent map with the buildings layer (top-right panel). In this 25$\times$25 km$^2$ tile we estimated a total area of 190 km$^2$ covered by flood water (30% of the total mapped area), potentially affecting 36,000 buildings. Nevertheless, many of the city buildings (shown in green) are not marked as flooded. According to the HRSL^27^, the estimated direct number of people affected in this scene is roughly 240,000. In the second row of Fig. 3, we show the results for the analysis in one of the grid tiles that is mostly covered by cropland, near the city of Layyah. At the right, we show the intersection of the cropland with the flood extent product. This analysis reveals a significant amount of water that overtopped the Indus river banks and inundated the crops covering the floodplain. In total, we estimated an area of 107 km$^2$ of cropland affected by flood water in this scene.

**Figure 4:**
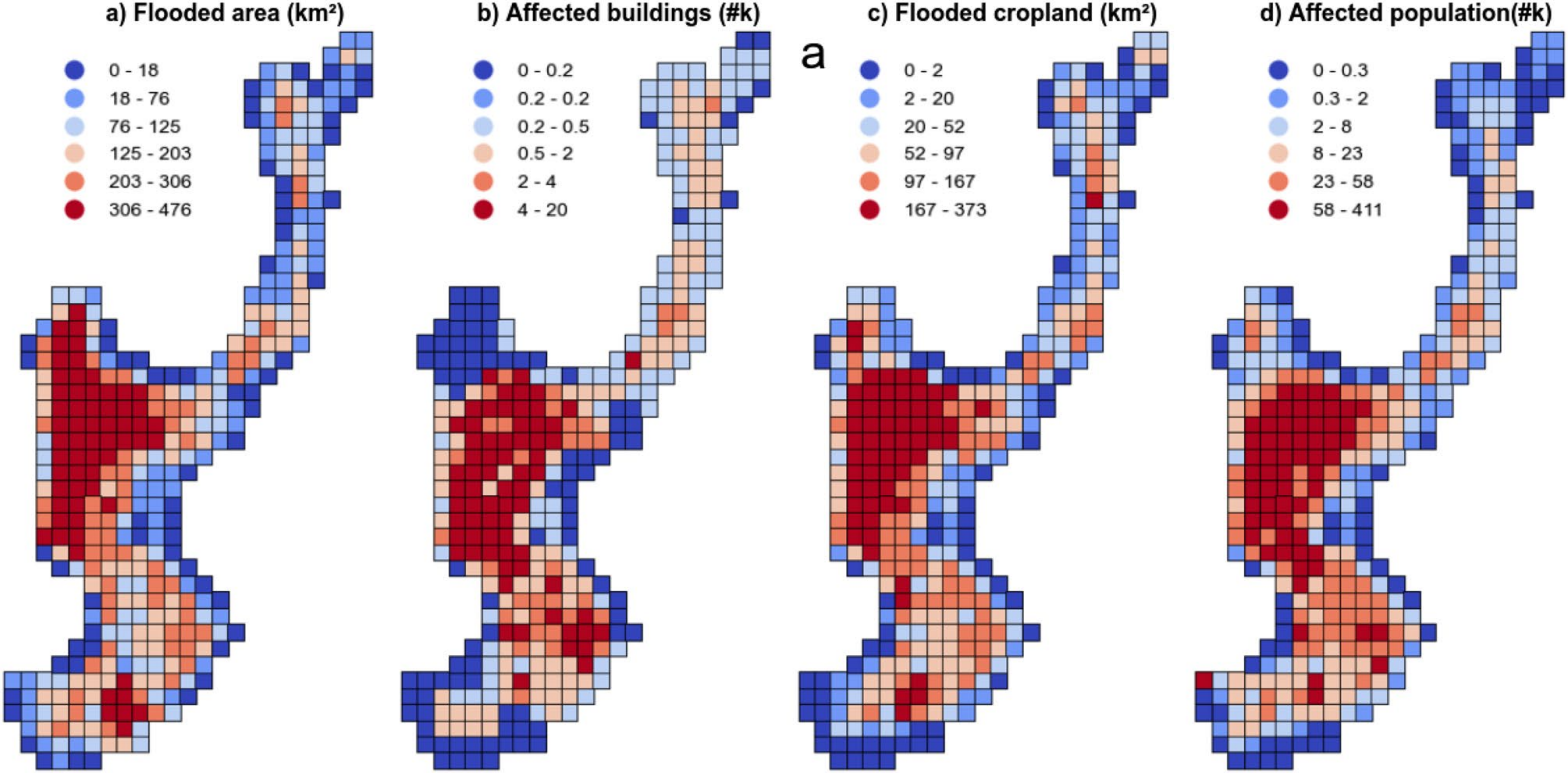
Local estimations of damage over the Indus basin. In total, 372 grid cells of 25$\times$25 km$^2$. From left to right, we show per cell estimations of: (**a**) area of water detected, (**b**) number of affected buildings, (**c**) area of flooded cropland, and (**d**) affected population counts.

We conducted this analysis for each of the 372 tiles of 25$\times$25 km$^2$ that encompass the mapped region of the Indus basin. Figure 4 provides an overview of the flooded area, affected buildings, flooded cropland, and affected population. The province of Sindh emerged as the most severely affected area, primarily due to its high population density and extensive cropland. Consequently, it suffered the greatest losses in terms of cropland, affected population, and buildings. Additionally, a substantial amount of floodwater was observed in Balochistan, near the Sindh border. However, this region has a lower population density, resulting in comparatively lesser estimated damage. Finally, the province of Punjab, located in the top-east region, also experienced a significant impact. The analysis suggests that an estimated total area of approximately 54,300 km$^2$ was covered by floodwater, encompassing approximately 28,700 km$^2$ of cropland. The flood event is estimated to have impacted around 10.8 million individuals, and roughly 656,000 buildings were potentially affected by flooding.

To provide a comparative perspective, we intersected the same building, cropland, and population data with the flood map released by UNOSAT^11^, generated using VIIRS at a spatial resolution of 375m. This analysis revealed that 43,300 km$^2$ were potentially covered by flood water, 25,700 km$^2$ of which would be cropland. Moreover, the flood event had an estimated impact on approximately 10.0 million people and the potential to damage a total of around 673,000 buildings. The statistics obtained from both flood products exhibit consistency, thus supporting the reliability of our method.

#### Australian floods

In 2022, the eastern part of Australia was affected by persistent heavy rainfall and successive extreme weather events that broke many historical records^8^. For instance, in two months from February to April the equivalent of one years’ average rainfall occurred in the New South Wales (NSW) region^33^. In Queensland, official sources counted tens of thousands of displaced people and around twenty thousand flooded homes.

We deployed our flood mapping system to generate a maximum-extent water map covering over 450,000 km$^2$, sampled by 763 processing tiles ($25\,$km on a side, as before). The total area of interest includes parts of southeast Queensland, eastern NSW and eastern Victoria, as illustrated in the top-left panel of Fig. 1. The time-range of interest spanned February 26th to March 30th, encompassing several successive rainfall events. In total, more 14,681 Sentinel-2 images and 9328 Landsat-8 images were curated to produce the final flood map. We followed an identical procedure as described previously to generate pre- and post-flood maps, and we estimated that approximately 27,000 km$^2$ were likely inundated, and by intersecting the derived products with Bing Microsoft Buildings maps we found that 125,00 buildings were potentially affected in the event, with significant impact on the area near Brisbane, in Queensland.

### Machine learning based flood extent segmentation

Flood extent delineation from multispectral satellite images is tackled in this work as a binary semantic segmentation task. We want to classify each pixel in an image as land or water (either flood water or permanent water). Most basic remote sensing techniques using optical data to detect water relied on spectral indices such as the Normalized Difference Vegetation Index (NDVI)^34^. After the inclusion of thermal and more infrared bands in MODIS and Landsat missions, specific water detection indices were designed, e.g. the Normalized Difference Water Index (NDWI)^19^ and a modified version (MNDWI)^20^ that uses the short-wave infrared (SWIR) bands to substantially improve water detection. These indices are fast and easy to compute, and one can obtain a binary segmentation mask by applying a threshold.

Deep learning methods such as Convolutional Neural Networks (CNNs) and Fully Convolutional Neural Networks (FCNNs)^35,36^ have shown superior performance in the task of semantic segmentation of remote sensing images in problems such as land-use classification^37^, cloud detection^38,39^ and also flood and water detection^16,40^. Contrarily to classical spectral methods, DL methods excel at discerning intricate structures and patterns unique to water in a multi-dimensional, often low-level, feature space. Concretely, FCNNs exploit both spectral and spatial dimensions of satellite images by learning these patterns from the huge catalog of EO data. However, the training process of deep learning methods requires a huge amount of labeled data, i.e. annotated reference masks that are fed into the network to learn the relevant features and the best mapping to classify water. This is a problematic limitation, since labeling satellite images is time-consuming, and requires significant expertise, especially to distinguish water bodies mixed with clouds and cloud shadows, which are frequent in flood events. Currently, only a few labeled datasets for flood extent segmentation are publicly available. On the one hand, the Sen1Floods11 dataset^41^ consists of 446 512$\times$512 patches of Sentinel-1, Sentinel-2, and manually labeled ground-truth masks, as well as 4370 automatically labeled patches that were extracted from 11 flood events. On the other hand, the WorldFloods dataset^16^ contains co-located pairs of Sentinel-2 images and reference flood extent masks from 119 flood events that occurred between 2016 and 2019, resulting in approximately 180,000 patches of size 256$\times$256. It is the result of harmonizing flood extent products that have been generated by emergency response organizations such as Copernicus Emergency Management Service (CEMS), UNOSAT, or GlofMIR when their services are activated to cover flood events. The size of this dataset allows the training of large deep learning models. However, it has some drawbacks such as the limited size of the test set, which hampers the evaluation of the trained models or the low quality of several flood maps in the training split.

**Figure 5:**
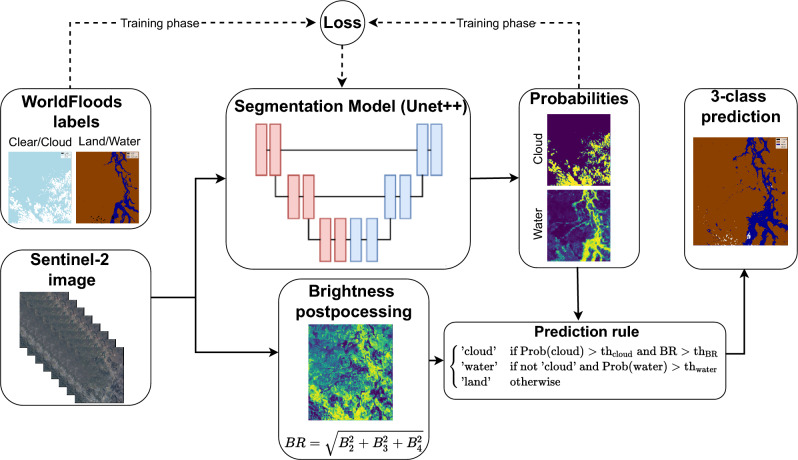
Multioutput binary model training and inference flowchart. The 2-layer reference masks corresponding to the cloud/clear and land/water problems is fed to train the segmentation model (in our best case, a Unet++). The input features are a C$\times$H$\times$W Sentinel-2 image patches, where C is the number of channels of the image. Once the model is trained, it produces an output with two layers, one that gives the per-pixel probability of cloud, and another that gives the per-pixel probability of water. The brightness reflectance of the input image is computed as the norm of the visible bands. The models’ output probability and brightness are then combined according to the prediction rule to yield the final flattened three-class segmentation mask.

In this work, we build a framework (i.e. data pipeline, dataset, models, and deployment platform) that can be applied operationally to produce flood extent maps in flood events from any location of the world. In order to build a globally diverse dataset we further improved and extended the WorldFloods dataset. Firstly, we incorporated the flood events that occurred from 2019 to 2023. Secondly, we filtered low-quality flood maps that were mostly covered by clouds or presented significant labeling errors. Thirdly, we manually curated more than 200 maps using a labeling tool specifically developed for this work. Finally, we created new validation and test sets with high spatial variability to ensure robust model selection and evaluation. The resulting dataset has 13-band Sentinel-2 level 1C patches co-located with their corresponding reference masks from more than 180 different flood events. In total, when we split the data in tiles of 256$\times$256 pixels, we get approximately 75,000 sub-images pairs (see Fig. 10 for more details).

Regarding the presence of clouds, they are usually frequent in peak-flood and post-flood imagery. Thick clouds force to discard many observations, and thin clouds are one of the main sources of classification errors and increased uncertainty in the predicted water mask^42^. Previous works^43^ developed models for Landsat that were forced to predict presence or absence of water underneath the clouds. We adopted a similar strategy albeit more conservative for the reference labels generation. Instead of one layer containing three, mutually exclusive classes (land, water, and cloud), we generated two independent binary reference masks, one for the clear/cloud problem and another for the land/water problem. These are two separate (but overlapped) problems, and a multioutput model can be trained to confidently distinguish many types of clouds (e.g. thick clouds and semitransparent clouds), and at the same time be confident at predicting land/water in cases where the clouds permit to partially see the ground. This allows the models to output the maximum amount of information and the user to decide to be more or less cloud conservative. Moreover, creating a separate binary mask for clouds, instead of grouping these in a non-water class, makes the learning easier than in previous studies^43^ and allows one to take advantage of newly developed cloud detection algorithms^44,45^ that have significantly increased cloud detection accuracy. We also included in the label generation process small water streams from CEMS flood maps which were not included in the first version of the dataset. Figure 5 shows at the top the training procedure were the two-layer binary masks are used to train the multioutput segmentation models.

**Figure 6:**
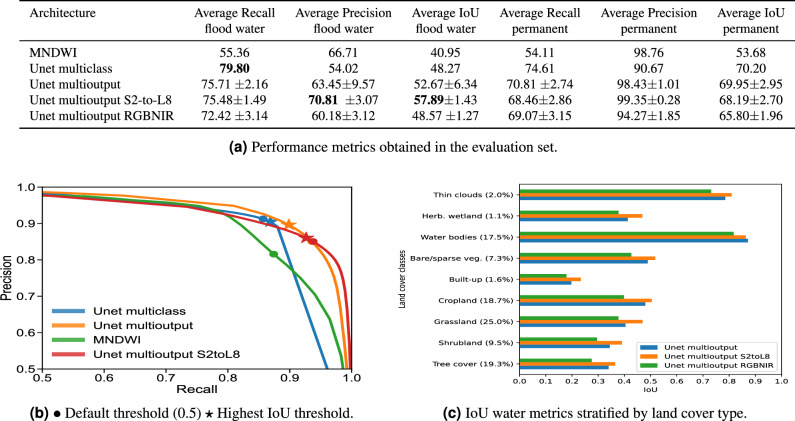
(**a**) Performance metrics obtained in the evaluation set, at a 0.5 threshold for water probability output from Unet models and a 0.0 threshold for MNDWI output. (**b**) Precision-recall (PR) curve for the best models and MNDWI (default threshold is 0 for MNDWI). (**c**) Mean IoU of the class water (flood) stratified per land cover type, according to ESA Land Cover classes. The percentage of each class in the dataset is indicated, and minority (or non existent) classes of Mangroves, Snow and Ice, and Moss and Lichen have been removed.

Once a segmentation model with a multioutput layer is trained (see "Methods" section for training details), an image of arbitrary size can be fed to output cloud and water probabilities of the same size. Afterward, we produce a three-class segmentation mask by combining the water and cloud probabilities with the brightness computed from the visible bands according to the prediction rule detailed in Fig. 5. The brightness threshold is included to distinguish between bright clouds were we assume surface cannot be observed and thin clouds. We set this threshold $th_{BR}$ experimentally to 0.35. The thresholds applied to the output probabilities to classify a pixel as cloud or water are $th_{cloud}$ and $th_{water}$, respectively. We set them to 0.5 by default, but it is possible to tune them according to the user requirements (depending on the application the user might need to be more or less cloud and water conservative). In order to let the users make an informed decision about the thresholds, Fig. 6b shows the Precision-Recall (PR) curve which illustrates the trade-offs of changing the water probability threshold. It can be seen that with the default threshold of 0.5 the multioutput model has a very high recall, which is good in order to detect most of the flooded areas. However, the threshold that resulted in the highest Intersection over Union (IoU) value in our evaluation dataset is 0.7, meaning that changing the threshold towards more restrictive values can result in a better balance for tasks where it is important to not commit false positives. In comparison, the original multiclass model from^16^ has less room for tuning the predictions, and changing the threshold in MNDWI would produce a high rate of false positives or false negatives.

### Global validation of proposed models

Before showcasing our model in Australia and Pakistan floods, we have validated our models in a novel benchmarking dataset that satisfies the required conditions for a robust evaluation. It is composed of 17 flood maps from 11 flood events that occurred in a wide variety of locations and biomes of the world, i.e. it is a global dataset (see Fig. 6) and it has been manually curated to ensure the trust in the evaluation results. We show the evaluation results of five models. As a baseline, we use the MNDWI^20^ with bands B11 and B3 of Sentinel-2. Additionally, we also consider a linear model as a second less strong baseline. Our proposed clouds-aware operational segmentation model is a modified Unet architecture that we call Unet multioutput, which uses Sentinel-2 as input to produce a segmentation mask. The main advantage of this multioutput model is that it produces two independent binary masks: one for cloudy/clear pixels and another for the water segmentation mask. Additionally, we refer to Unet S2-to-L8 for a modified version that has been trained with Sentinel-2 visible bands, NIR band (B8), and SWIR bands, resulting in a model that can also be applied to Landsat 8 images. Finally, we compare these models with an Unet multiclass that was originally proposed in ref.^16^, which produces a segmentation mask of three mutually exclusive classes: land, water, and clouds. With regard to the evaluation metrics, we considered recall, precision, and IoU for the water class: three common segmentation metrics that highlight the possible errors that can be committed in binary segmentation. Values are computed for each flood event and we report the mean value across all floods in the test dataset (see performance metrics in Fig.  6). For further comparison of the generalization of the models at detecting flood water, we calculated the IoU of the water class stratified by land cover classes according to the ESA Land Cover (Fig. 6c). We also analyze a “Thin cloud” class, generated by combining the cloud labels from the reference mask with the brightness threshold of 0.3. We refer the reader to the "Methods" section and Supplementary material for more details about evaluation metrics, the dataset, models, and training strategy.

MNDWI has been shown to be a strong baseline. It produces fairly good results in terms of accuracy, taking into account that it was applied with a general threshold for all flood events. However, in our experiments, deep learning segmentation models outperform MNDWI results in terms of all considered metrics.

Multioutput models have higher IoU than multiclass model when detecting flood water, which is arguably the hardest and most important task. Apart from the metrics, the output layer of these models is highly configurable and can be tuned to yield a model with more restrictive predictions in terms of water detection. We provide evidence of the advantages of using these models in Fig. 8 and Supplementary Figs. 3 and 4.

Regarding the stratification by land cover type, we observe an overall good performance of the models for the majority of classes, with lower performances in built-up and forest areas. Due to the high spectral mixing that exists in these areas, it is expected that detecting flood water becomes more challenging. The results of our analysis are consistent with other studies, which also reported lower performances in built-up and forest areas^15^. Among multioutput models, Unet S2-to-L8 is consistently the best-performing model. It is also worth mentioning that has lower variance across different training initializations. It uses the most important bands for water detection, i.e. visible, NIR, and SWIR bands, and we suspect that adding additional Sentinel-2 bands to the model only adds redundant information. Consequently, addressing this complexity requires a higher-dimensional model, which, in turn, necessitates a larger training set and a longer training period to converge to a solution and can become more dependent on the initialization. In addition to the quantitative results, this model also offers a huge operational advantage, since it can be applied to both Sentinel-2 and Landsat 8/9 images. Finally, it is worth mentioning that the accuracy of models without the SWIR bands (i.e. Unet multioutput RGBNIR) does not decrease significantly. Supplementary Fig. 1 shows the predictions of models that use different band combinations.

**Figure 7:**
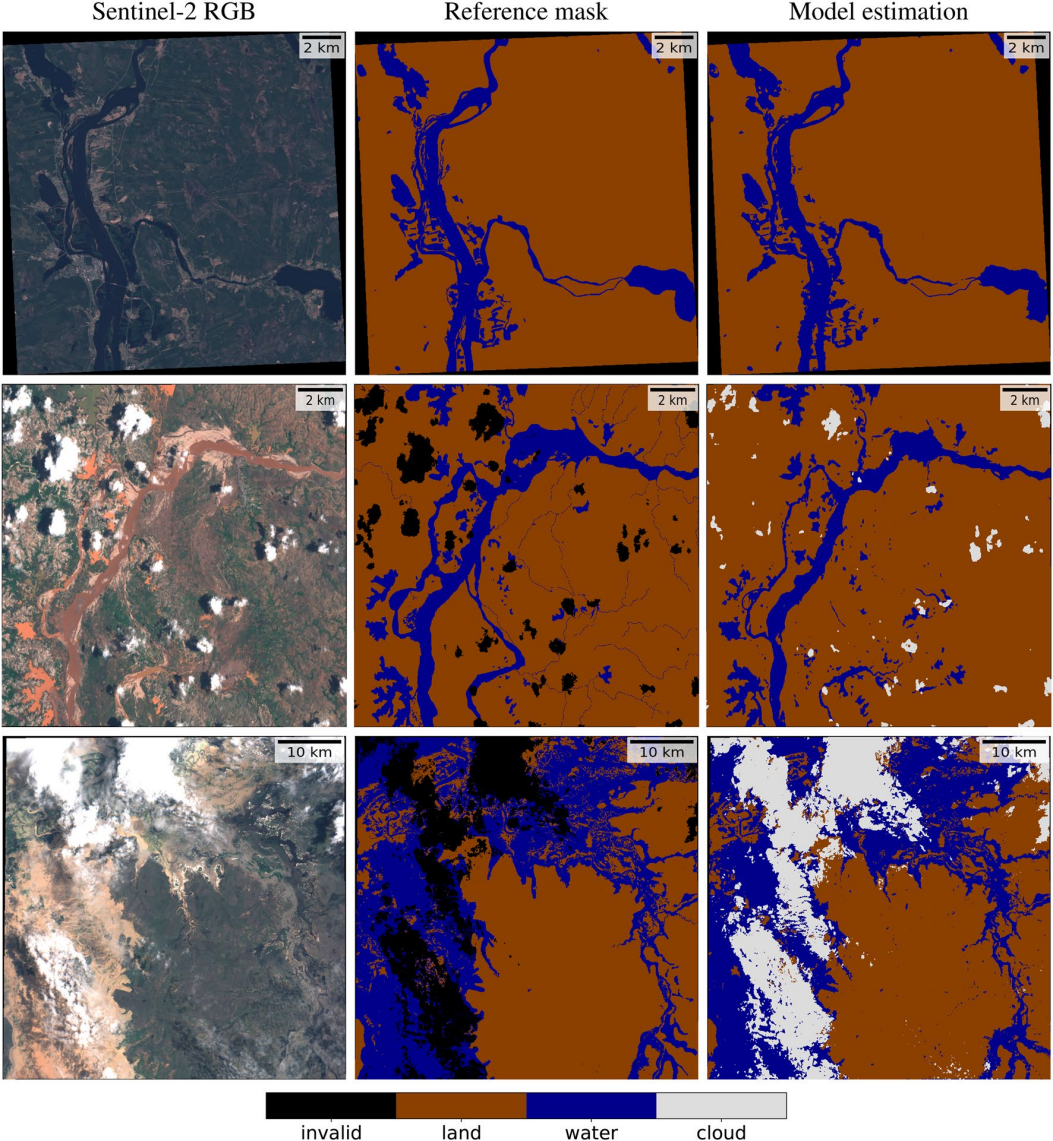
Multioutput binary model predictions in evaluation images. From top to bottom: flood events in Finland, Madagascar, and Australia. These are meant to represent models’ performance in different real-case scenarios of post-flood imagery in a variety of worldwide locations: a post-flood cloud-free image at peak-flood time, a cloud-free image with presence of flood traces, and a moderately cloudy image with presence of flood water covered by thin clouds, respectively.

Figure 7 shows the predictions generated by the Unet multioutput S2-to-L8 in three flood events that are part of our global evaluation dataset. In particular, they correspond to floods in Finland, Madagascar, and Australia. Firstly, the event in Finland shows a cloud-free image that was acquired at the peak of the flood. This is a relatively easy case to predict, and the model accurately captures all the water. Secondly, the event in Madagascar shows post-flood imagery that has not been acquired at peak time; some areas are covered by mud and water debris, i.e. flood traces. In this case, our model is able to capture both the flood water and most of the flood traces, which can be really useful in post-flood damage quantification when the first satellite overpass is not coincident with the flood peak, or images are overly cloudy. Finally, the Australian evaluation event shows a moderately-cloudy image with plenty of flood water. In this type of images, cloud shadows can be challenging, as well as cases where there is water under thin clouds.

**Figure 8:**
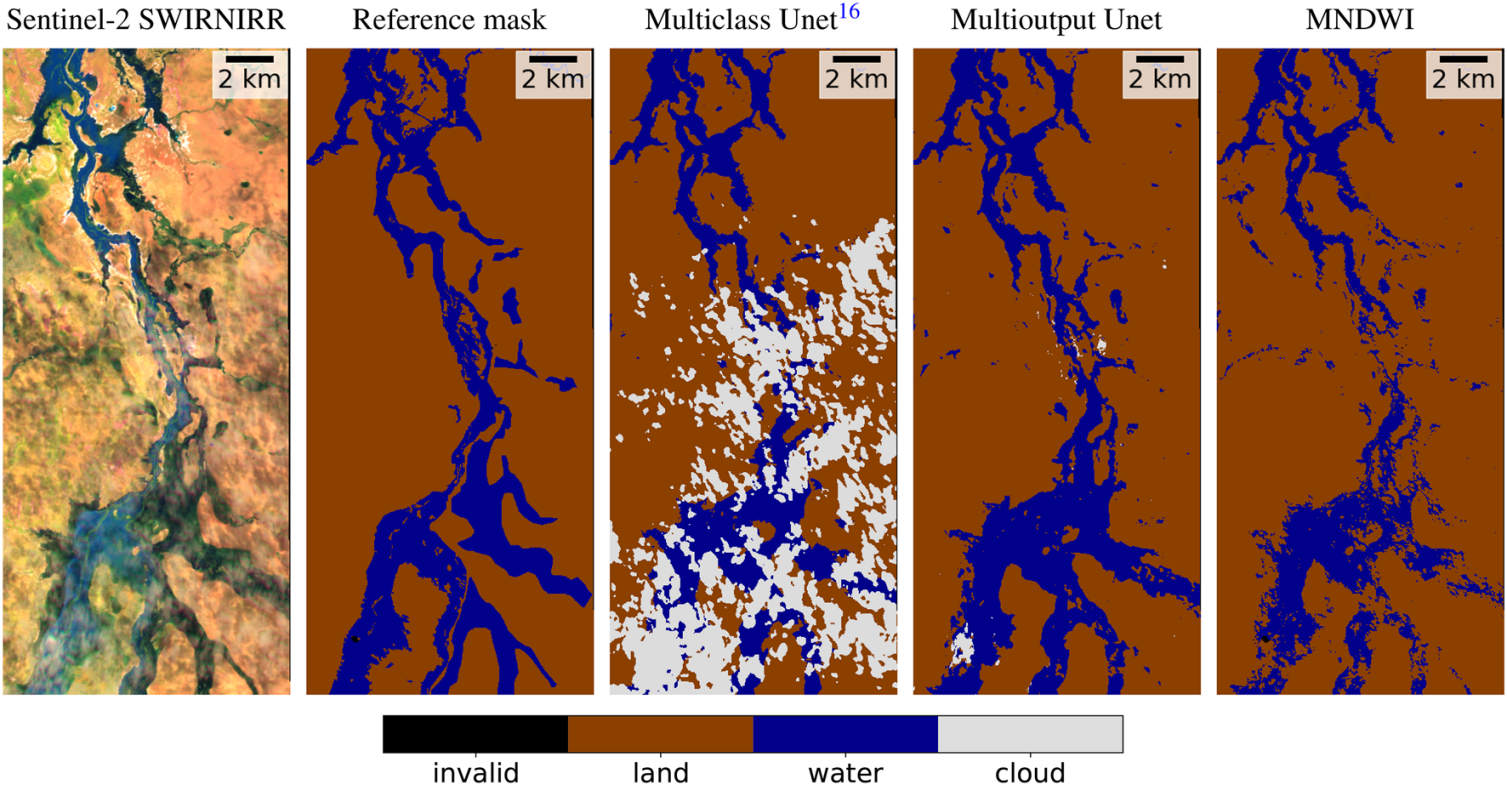
Predictions for an evaluation flood event in Australia, in an area with presence of thin clouds and flood water. The multioutput Unet correctly delineates the flood extent even in presence of semitransparent clouds. MNDWI is hampered by clouds and predicts much less flood extent than the actual one, while multiclass Unet discards a lot of valuable information by predicting ‘cloud’ in thin clouds where the flood water can be distinguished.

Since one of the biggest advantages of our model is being able to account for or discard observations under thin clouds, we have zoomed in on an area of the Australian event (Fig. 8) to compare the predictions generated by all the models in this type of scenario. First, a false color Sentinel-2 composite that highlights water is shown (bands 12, 8, and 4), followed by the reference segmentation mask and the predictions generated by the three main models that we are comparing. The multiclass Unet produces highly accurate predictions in cloud-free areas (top of the image), however, predicting three mutually exclusive classes results in thin clouds being classified as clouds and thus discarding valuable information that can actually be extracted from the acquired image. The segmentation mask produced by the multioutput Unet only predicts ‘cloud’ over thick clouds, which does not allow confident detection of flooded areas, while showing accurate predictions in cloud-free areas and flooded areas under semitransparent clouds. Finally, MNDWI produces moderately accurate predictions in cloud-free areas but fails in regions surrounded by thin clouds (which are combined with land and any non-water surface).

**Figure 9:**
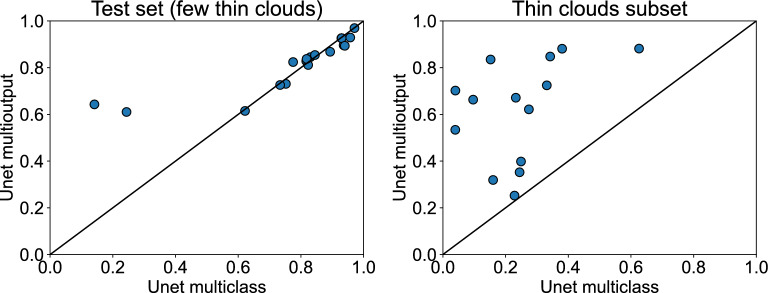
Comparison of models in images with thin clouds. We compare the IoU obtained by the multiclass and multioutput model in two evaluation sets: one with few thin clouds (left) and another with higher presence of thin clouds (right).

**Figure 10:**
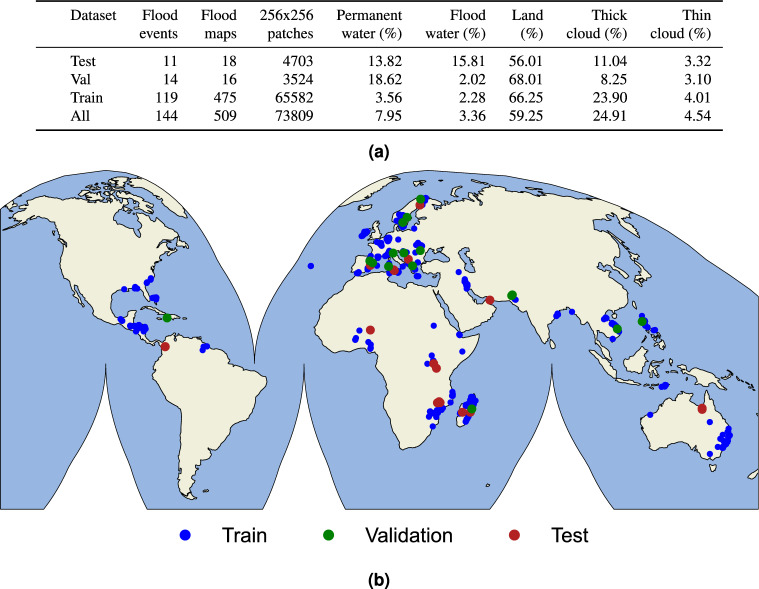
(**a**) Statistics of the extended WorldFloods dataset used to train the models. (**b**) Locations of the flood events included in the extended WorldFloods dataset. Color codes correspond to the associated data split for training and validating the models.

To further extend the visual analysis of the predictions in presence of thin clouds, we have created an additional evaluation set composed of WorldFloods events that contain a high percentage of transparent clouds and water. In this dataset, we have compared the IoU of multiclass and multioutput Unet (Fig. 9). In the plot of the left, we see that in events where there are very few thin clouds both models perform similarly in most of the events. However, in events with abundant semitransparent clouds (right plot), the multioutput model exhibits significantly higher IoU than the multiclass model. We also see in this figure that the performance of the models in these scenes with thin clouds is lower. This shall be taken into account for operational applications: for instance, if the image has many thin clouds, these scenes shall be validated by human experts before utilizing them to produce the final flood extent product, regardless of the selected method. Nevertheless, thin clouds represent a total amount of 4.54% of pixels in the WorldFloods dataset (see Fig. 10a); to compare with, flood water represents 3.36% of the dataset. Considering the trade-offs, with the proposed model we increase the amount of usable post-flood imagery leading to more precise flood extent maps. Additionally, to provide a deeper exploration into the ramifications of the brightness (BR) threshold adjustments on the model’s predictive capabilities, we have computed the Precision-Recall curves for different values of the brightness threshold, both in WorldFloods test set and in the thin clouds subset. These results can be found in supplementary Fig. 5. Moreover, Fig. 4 shows a visual demonstration of the produced segmentation masks, showcasing the impact of adjusting the brightness threshold in flood maps with a significant presence of thin clouds. The effect of changing the brightness threshold is minimal in the test set since there are very few examples of thin clouds in comparison to thick clouds or clear observations. However, from the results obtained in the thin clouds subset it can be seen that increasing the brightness threshold results in lower precision. This is expected, because increasing the threshold causes more (thick) cloudy observations to be treated as thin clouds, thus being included in the predicted water masks, likely resulting in more misclassifications. From our experiments in flood segmentation, empirical evidence running the models in inference mode, and previous work in cloud segmentation^46^, we concluded that 0.3 is a reasonable brightness threshold to fully exploit the available observations without sacrificing precision. However, if one would like to mask all these observations, the threshold could be reduced to 0. In this case, the output cloud mask would be completely determined by the output of the cloud segmentation model.

## Discussion

In this work, we have presented an end-to-end pipeline for flood extent mapping in multispectral satellite images that can be used operationally at any location in the world. We have presented a detailed validation of its robustness and global applicability in a manually labeled data set composed of 11 flood events that occurred in a wide variety of locations and biomes.

One differentiating factor of our proposal is that it can deal with the presence of thick and semitransparent clouds, which are frequent in post-flood imagery. Previous works exclude partially cloudy images from the training or inference steps^14^. In our view, this has several drawbacks: firstly because some recent works have found that training deep learning models on cloud free imagery causes severe misclassification problems^42^. Secondly because excluding cloudy images leads to an under-exploitation of the available information which is critical in emergency response scenarios.

Our proposed methodology is also configurable and it can be tuned depending on the needs and available imagery. For instance, if a conservative estimation of water extent is needed, the brightness threshold can be adjusted to discard areas covered by semitransparent clouds (see Supplementary Fig. 4). Additionally, due to the temporal misalignment that exists for some samples of the dataset, the models detect also flood traces (areas that are not inundated in the current scene but have been in the past). This can be useful in scenarios when there is limited post-event data. If those are not needed, flood traces can be discarded by adjusting the water threshold (see the PR curve on Fig. 6) or by combining the output with the MNDWI.

The proposed models have been trained on different bands combinations and they can be applied to Sentinel-2 but also Landsat 8 and 9. As a result, the output products have a spatial resolution of 10m to 30m, much higher than the resolution utilized in other large scale flood mapping works (e.g. Refs.^4,11,47^ which use MODIS or VIIRS data). Also, this interoperability increases the temporal cadence of images, allowing to have images almost daily, which is key for monitoring the evolution of the floods. It can also enable the detection of short-time events that might be undetected with larger temporal revisits^13^.

We have tested our methodology in two large-scale flood events that occurred in 2022 in Australia and Pakistan. In Pakistan, the derived statistics are consistent with the analysis carried out for the flood map released by UNOSAT, which was produced with VIIRS imagery of 375 m resolution. Although this results are encouraging, further validation is required in the process of deriving the metrics of the flood impact. In particular, there are several uncertainties that affect the estimates of affected population. On the one hand, land cover classification in urban areas is challenging, even with optical satellites, due to the heterogeneity of background^15^, and the flood extent delineation problem is not an exception^48^. On the other hand, estimates of flood exposure present high discrepancies depending on the population density data that is used^10^.

We foresee two main directions of future work. Firstly, by utilizing the proposed pipeline, the impact of past flood events can be re-quantified. This can be helpful, for instance, to study flood-induced migrations, as well as flood exposure^49^. Secondly, by incorporating data of different modalities (e.g. SAR or DEM), we could increase the coverage on areas with persistent cloud coverage. Following this line an easy to do extension would be to add simultaneous Sentinel-1 imagery to our database. This expansion could enable the development of new strategies that overcome the limitations posed by cloudy optical scenes while maintaining the accuracy of the models in cloud-free scenarios. Another limitation to be tackled is the slightly worse performance of the models working only in RGBNIR bands. This will enable the applicability of the model on most commercial satellites which lack SWIR bands. This will increase, in turn, the availability of post-flood imagery. As shown in several works, SWIR information is crucial for accurate water detection^50,51^and there are already preliminary work aiming to mitigate this downside tested on commercial satellites imagery from Planet^52^.

## Methods

### Architectures and model training

In the updated version of the WorldFloods dataset, validation and test data split were carried out by hand-picked examples to evenly cover worldwide locations and biomes. The training set is composed of pairs of Sentinel-2 images and reference masks of the flood maps. However, we pay careful attention to avoid any data leakage by excluding from training the flood maps of events that are represented in test and validation sets. For instance, one flood event represented in the test split is EMSR342 (according to Copernicus EMS codes). Two of the flood maps corresponding to this flood event (06NORTHNORMANTON and 07SOUTHNORTHNORMANTON) are included in the test set. Therefore, we excluded from the training process the rest of the flood maps of this emergency activation.

The training dataset includes data from 475 flood maps of 119 different flood events (see Fig. 10b). Each flood map is split into patches of 256$\times$256 pixels to train the models. On each training epoch, we loop randomly over all the patches; in total there are around 65,000 256$\times$256 image patches in the training dataset. We trained the models using different band combinations as inputs. Unet multioutput is trained with all 13 bands of Sentinel-2. Unet S2-to-L8 is trained using the RGB, NIR and SWIR bands of Sentinel-2 (6 bands in total), which allows using of the models in Landsat 8 images. Finally, we also trained models using only RGB and NIR bands as inputs. With respect to the data augmentation, we used common nondestructive transformations such as flips and 90-degree rotations. The models produce as outputs two images: the water probability and the cloud probability (see Fig.  5). The models are trained to minimize the average binary cross-entropy loss over all the pixels on both problems (water/land and cloud/clear). The final loss weights the water/land problem higher (0.8) than the cloud/clear (0.2). The dataset has a strong class imbalance, being land much more frequent than clouds and water. We accounted for that by weighting the pixels of the binary-cross entropy loss for the water/land problem with the inverse frequency of the water class (Fig.  10a). This allows us to obtain high recall models, especially for the class of water which is the most relevant in flood detection. Finally, the learning rate was set to 10$^{-4}$ with a scheduled reduction after two epochs. We also implemented early stopping after 4 epochs if there was no improvement in validation loss, in order to avoid overfitting. All the necessary code to reproduce the training is implemented in our Python package ml4floods, available at https://pypi.org/project/ml4floods/.

### Metrics calculation

In order to evaluate models performance we have considered Precision, Recall and Intersection over Union (IoU), three common metrics in semantic segmentation problems. These are defined based on the possible errors that can be committed in a binary segmentation problem: (1) True Positives (TP), water pixels correctly classified as water; (2) True Negatives (TN), non-water pixels correctly classified as non-water regions; (3) False Positives (FP), non-water pixels incorrectly classified as water; and (4) False Negatives (FN), water pixels incorrectly classified as non-water. Precision measures the percentage of the TP pixels that are actually water, in contrast, recall measures the percentage of TP that have been captured by the model. Finally IoU is a trade-off between precision and recall.

1 $$\begin{aligned}&\text {Precision} = \frac{TP}{TP+FP}, \end{aligned}$$

2 $$\begin{aligned}&\text {Recall} = \frac{TP}{TP+FN}, \end{aligned}$$

3 $$\begin{aligned}&\text {IoU} = \frac{TP}{TP+FP+TN}, \end{aligned}$$

We compute these metrics for each flood map in the test and validation dataset masking invalid pixels and bright clouds (pixels that are labeled as clouds and have a brightness above 0.3), since the surface information in this case is totally blocked.

Figure 6c and Supplementary Table 1 report these metrics calculated over the water class in our evaluation dataset.

### Duplicate publication statement

Preliminary results of this work have been presented in AGU Fall Meeting 2021^17^ and ESA Living Planet Symposium 2022^18^. These conferences do not have conference proceedings and only accepted abstracts are published.

### Supplementary Information

Supplementary Information.

## Data Availability

WorldFloods extended flood maps and Pakistan flood maps can be found at Zenodo (https://zenodo.org/record/8153514). Satellite imagery from Landsat and Sentinel has been downloaded from the Google Earth Engine^53^.

## Code availability

All the necessary tools for ingesting data, training and evaluating models, and running inference in new data can be found in the ml4floods repository https://github.com/spaceml-org/ml4floods. Manuscript figures were created with our own custom software. Our software is open-source and uses other open-source python libraries for plotting.
