# Supplementary material for: Global flood extent segmentation in optical satellite images
Source: Sci Rep. 2023 Nov 20;13:20316. doi: 10.1038/s41598-023-47595-7 (PMC10661555; doi:10.1038/s41598-023-47595-7)
Supplement: Supplementary file 1 — Supplementary Information. [file 41598_2023_47595_MOESM1_ESM.pdf]

## Appendix: Supplementary material

**Table 1.** Performance metrics for all experiments. The values shown correspond to the 0.5 threshold for water for the multiclass and multioutput models. For MNDWI we set the threshold at 0. We observe that models trained in the extended dataset perform better. Additionally, we see that multi-output models have lower IoU than multiclass models but significantly higher recall.

| Architecture | Model version | Dataset | Mean recall per flood | Mean precision per flood | Mean IoU per flood |
|---|---|---|---|---|---|
| Unet | Multiclass | Extended | 92.23 | **91.51** | **85.29** |
| Unet | Multiclass | Original | 90.68 | 90.73 | 82.55 |
| Unet | Multioutput binary | Extended | 94.19±3.61 | 86.94±2.50 | 82.45±2.24 |
| Unet S2-to-L8 | Multioutput binary | Extended | **96.35**±0.37 | 86.18±1.22 | 83.49±0.88 |
| Unet RGBNIR | Multioutput binary | Extended | 93.50±1.43 | 84.33±2.57 | 79.86±1.74 |
| SimpleCNN | Multioutput binary | Extended | 96.27 | 73.17 | 71.01 |
| MNDWI | - | - | 85.87 | 80.56 | 70.45 |
| linear | Multioutput binary | Extended | 87.06 | 73.27 | 64.62 |

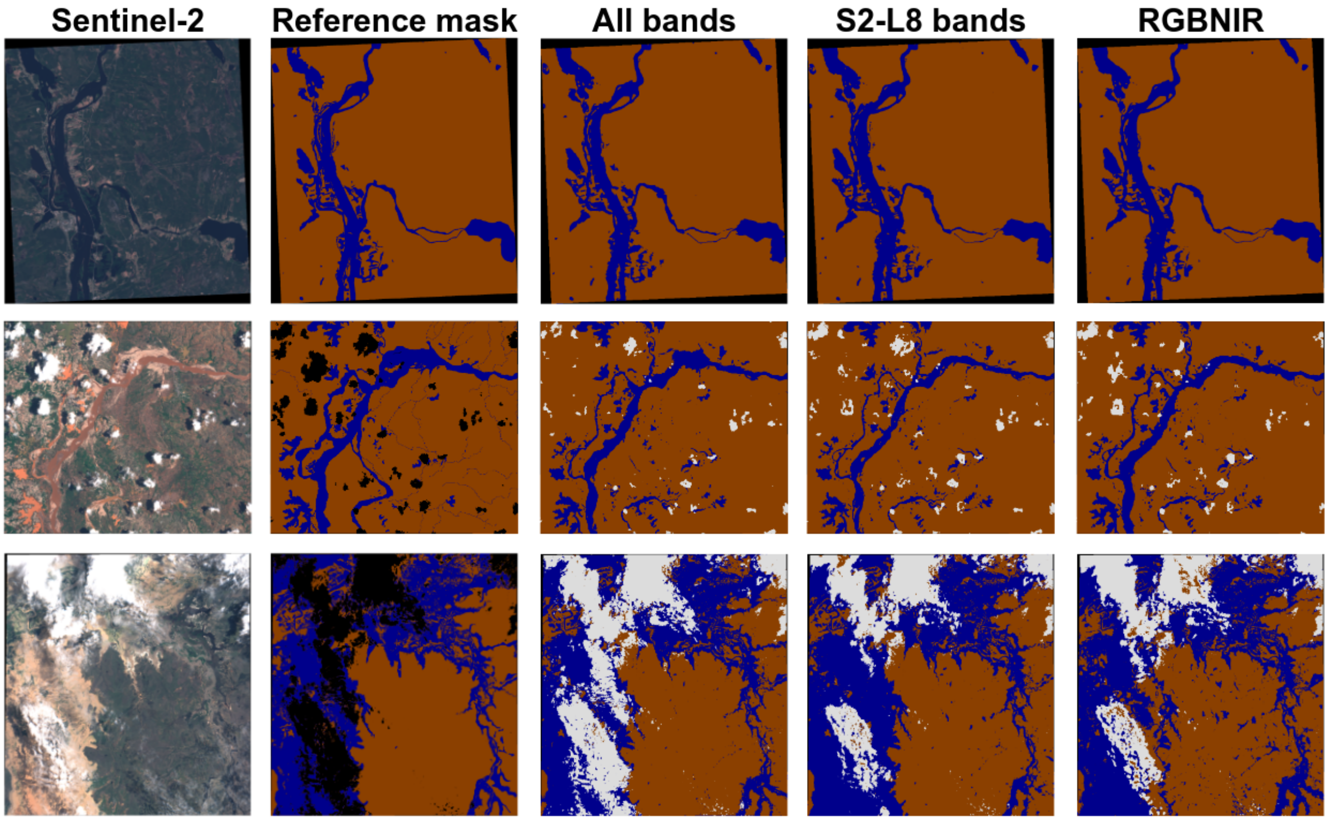


**Figure 1.** Multioutput predictions for different channel configurations: c) Sentinel-2 channels (13 bands); d) RGB, NIR and SWIR (6 bands); e) RGB and NIR bands (4 bands).

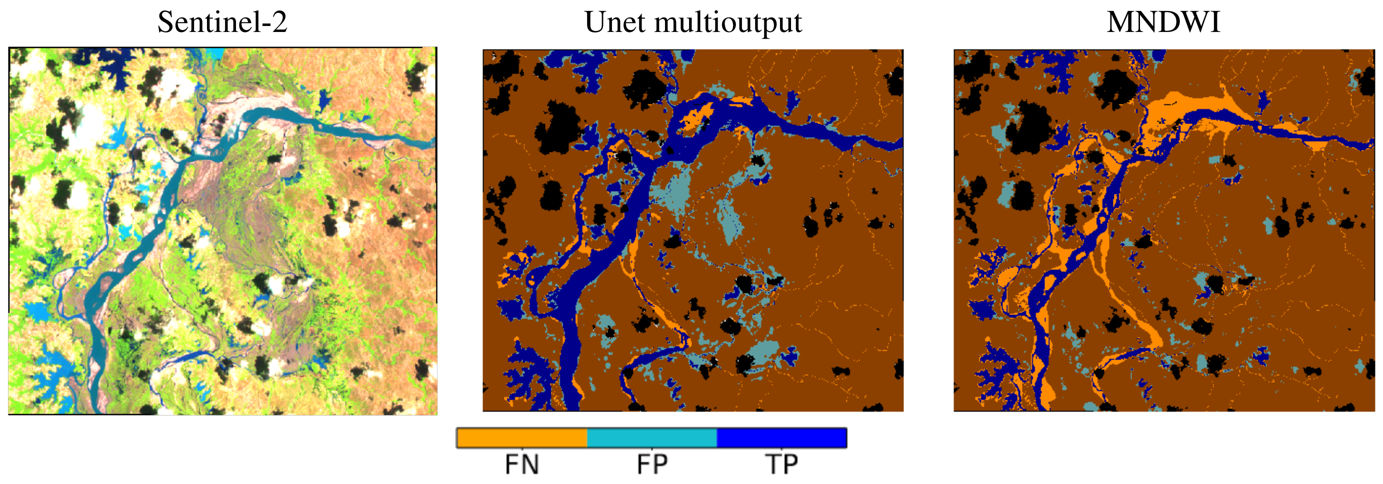


**Figure 2.** Error type comparison. The image on the left shows a Sentinel-2 false color composite (SWIR-NIR-Red bands) from the test set. Predictions of Unet multioutput S2-to-L8 (middle) and MNDWI (right) are shown, indicating False Negatives (FN), False Positives (FP) and True Positives (TP). Clouds and invalid pixels have been masked (shown in black). Both predictions correctly detect flood water areas, but MNDWI shows a great number of FN in areas covered by muddy water, i.e. flood traces. It also shows a high FP rate in cloud shadows. Instead, our model is able to detect most of the flood traces, and correctly predicts land in cloud shadow areas.

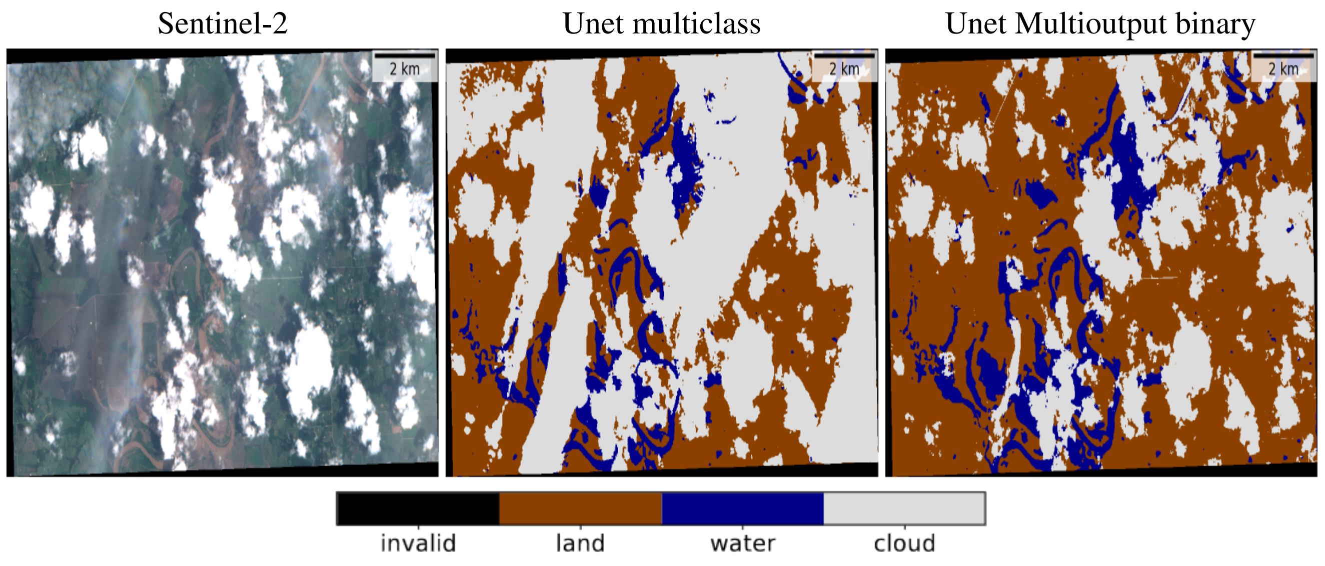


**Figure 3.** Predictions from the multiclass and multioutput binary models in a flood map from the thin cloud subset, corresponding to a flood event occurred in Brazos River (USA). The multioutput binary Unet is able to detect flood water under thin clouds, only predicting thick clouds. Contrarily, the multiclass model predicts much less water extent.

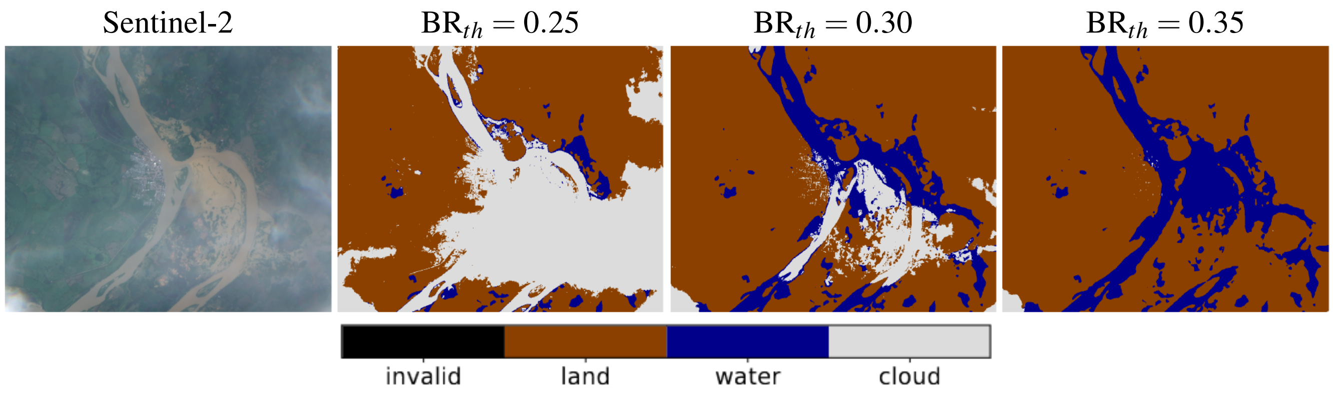


**Figure 4.** Multioutput Unet predictions for activation 264NECHI, for different values of the brightness threshold ($br_{th}$). By modifying this threshold it is possible to detect the water extent covered by thin clouds.

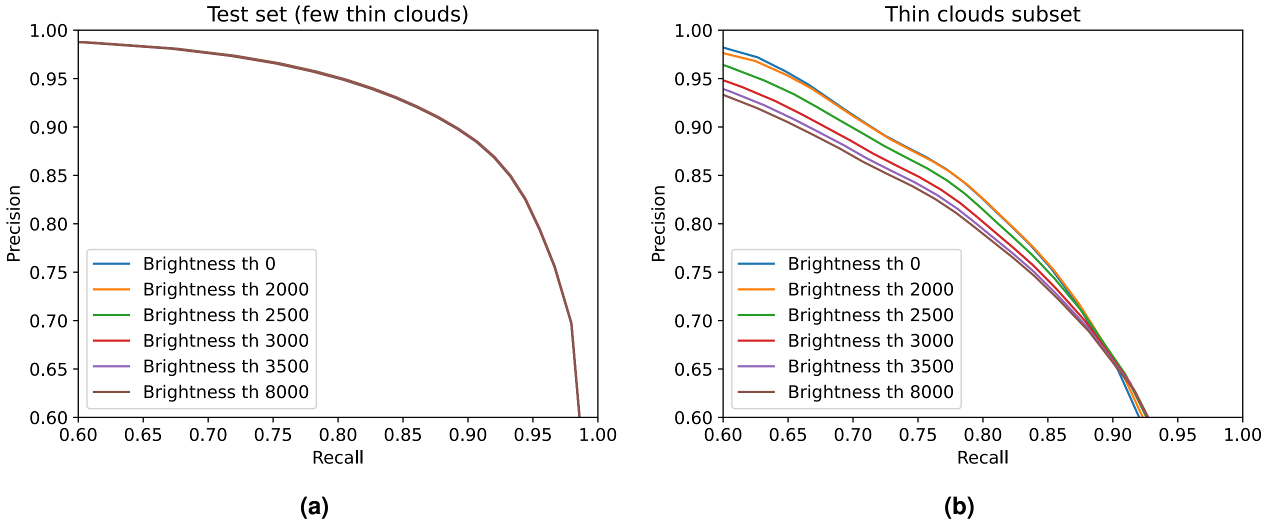


**Figure 5.** Precision-Recall (PR) curves for different brightness thresholds. (a) Calculated in WorldFloods test set. (b) Calculated in the thin clouds subset.
